# Traumatic penile amputation in a 15‐year‐old boy presenting late in northwestern Nigeria

**DOI:** 10.1002/ccr3.629

**Published:** 2016-07-11

**Authors:** Muhammad Ujudud Musa, Umar Farouk Abdulmajid, Sharfuddeen Abbas Mashi, Bashir Yunusa

**Affiliations:** ^1^ Surgery DepartmentFederal Medical Centre KatsinaKatsinaNigeria; ^2^ Surgery DepartmentAminu Kano Teaching HospitalKanoNigeria

**Keywords:** Amputation, penile, reimplantation, traumatic

## Abstract

Traumatic penile amputation is a serious urological emergency, although rare whenever it happens, there is a need to refer the patient early to urologist within 24 h, with the stump wrapped in saline; unfortunately, our patient presented late and as such could not benefit from penile reimplantation.

## Introduction

Posttraumatic penile amputation is a urological emergency, which can be due to self‐infliction as seen in psychiatric patients responding to command hallucination, or it can be from industrial accidents or assaults, and rarely from circumcision mishaps, especially guillotine type 1, 2, 3.

Penile amputation can be devastating to the patient, his spouse, and his relatives, and very tasking to the urologist, especially when the patient requires reimplantation of the penis and the stump was brought late after 24 h, and unpreserved, as is the case of our patient.

The blood loss can be severe requiring resuscitation with intravenous fluids, and blood transfusion may be necessary in some patients. In shocked patient, there will be the need to control active bleeding and adequate analgesia is necessary because of pain 4, 5.

The anatomical location of the penis makes it vulnerable to trauma, and the fact that men are more likely to participate in physical and contact sports, violence, and exposure to industrial accidents, hence about one‐third of the two‐third cases of genitourinary trauma affects the penis 6, 7.

The first successful penile reimplantation was reported by Ehrich in 1929, and there are different outcomes of treatment and success rate. There may be some remaining consequences, such as skin necrosis and urethral stricture or fistula, and the treatment also depends on the severity of the lesions, delay in presentation, and the patient's mental state 8, 9.

Traumatic penile amputation from grinding machine injury is a rare problem in our environment, and whenever it happened it occurs while the patient operates the machine and is wearing inappropriate cloth. This had what exactly happened to our patient 10.

## Case Description

A 15‐year‐old boy who was referred from a peripheral hospital, with a history of penile amputation following trauma from a grinding machine which he was operating, was said to have sustained traumatic penile amputation, with severe bleeding and pain, and no history of loss of consciousness.

He was initially taken to the local hospital, where he was resuscitated and the bleeding vessels ligated; the amputated part of the penis was wrapped in gauze before he was referred to us and he presented to us about 30 h after the incidence.

On physical examination, he was found to be anxious mildly pale not dehydrated. There was a complete penile amputation through his penile skin to the cavernosal bodies and transaction of the urethra with bleeding from the dorsal vessels. His scrotum was lacerated exposing the testicles with devitalized tissues; however, the testicles were found to be intact with a spigotted catheter and the mummifying penile stump in between his thighs.

**Figure 1:**
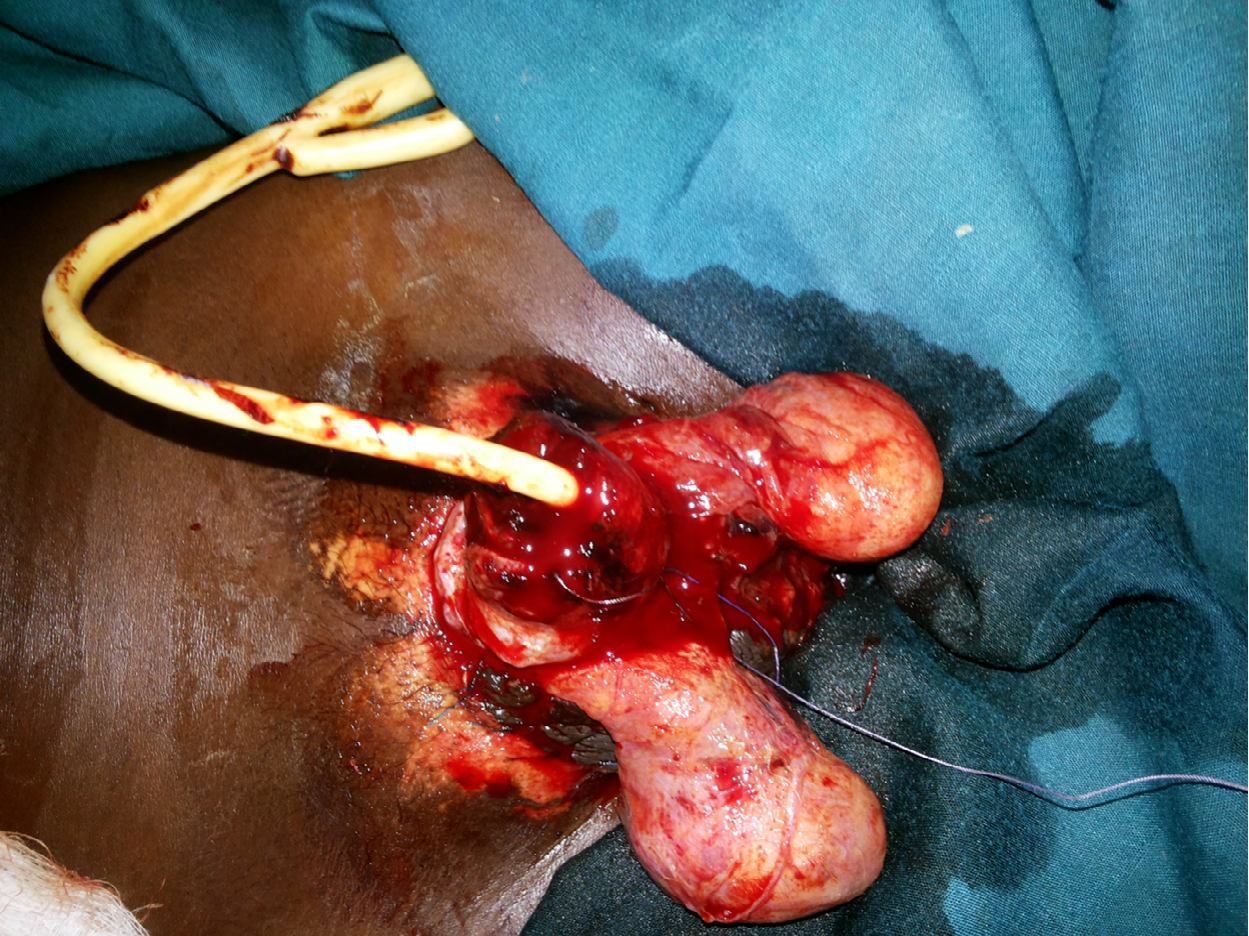
Posttraumatic penile amputation stump after debridement and Hemostasis.

After thorough irrigation with normal saline, parenteral antibiotic and analgesic was given. He was transfused with two units of whole blood and was taken to the theatre under general anesthesia. A rubber band was placed, as a tourniquet, around the proximal end of his penis for bleeding control and debridement was done, and the bleeding vessels were identified and ligated as shown in Figure 1.

**Figure 2:**
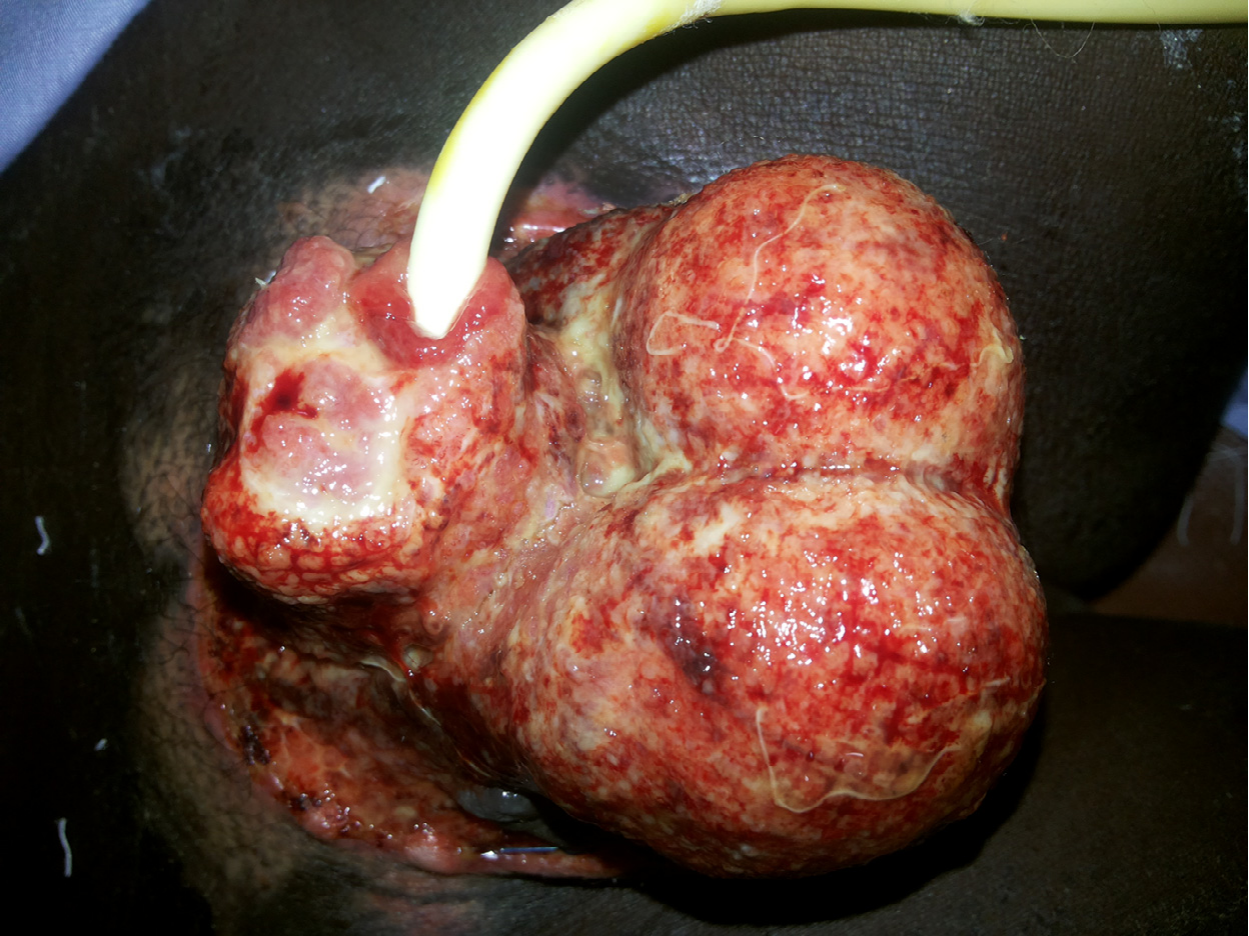
Five days post debridement with wound granulating.

A 16Fr Foleys catheter was inserted transurethrally through the urethral stump followed by everting the urethral edges and suturing it using interrupted 4/0 vicryl sutures. The wound was dressed with Vaseline gauze and was found to be granulating well 5 days postoperatively as shown in Figure 2.

**Figure 3:**
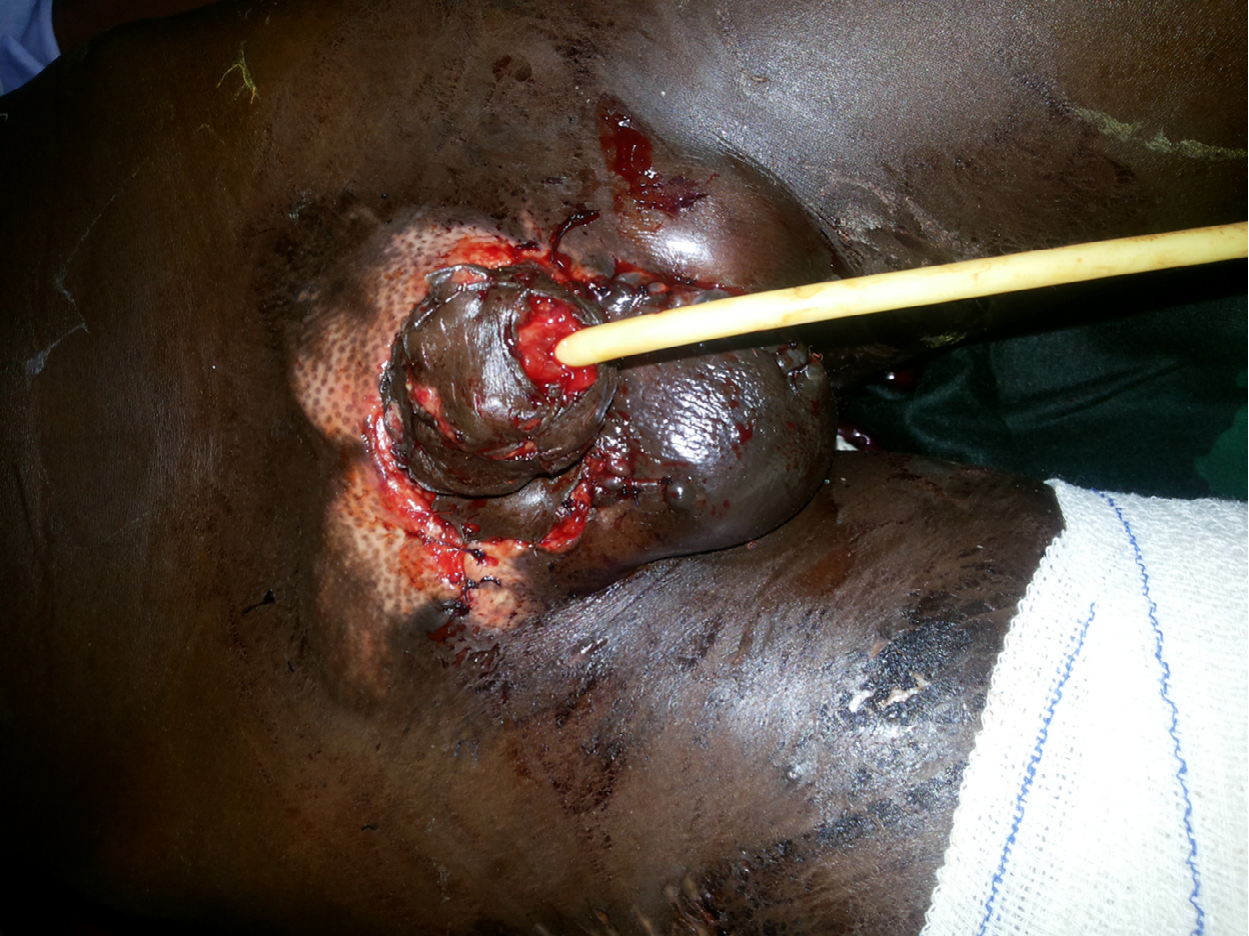
After skin grafting and scrotoplasty.

Eight days postoperatively, patient was prepared and had skin grafting using a split thickness graft from the thigh skin of the patient. The edges of the graft were sutured with vicryl 4/0, and the testicles were returned to the scrotum and scrotoplasty was done as shown in Figure 3.

**Figure 4:**
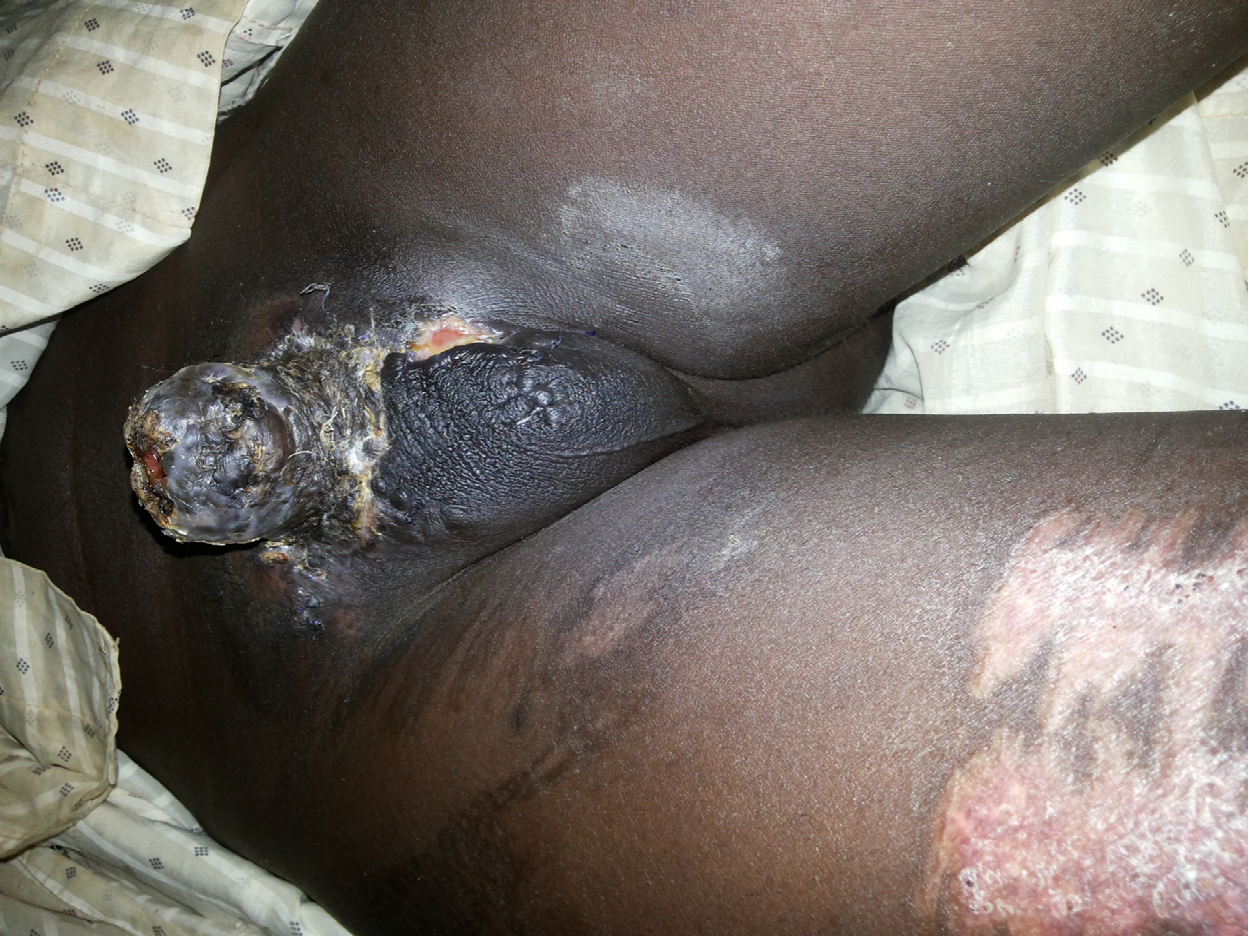
Weeks post‐split thickness skin grafting.

Four weeks post‐split thickness, skin grafting patient did very well, wound had healed and he was able to pass urine with good flow. On follow‐up examination, 5 weeks later, no necrosis was noticed on his skin Figure 4.

## Discussion

The causes of penile amputation includes industrial trauma, as is the case of our patient, or road traffic accident, and in some patients, from gunshot injury or other penetrating injuries. Other patients presenting with penile amputations are due to self‐mutilation, especially psychiatric patients or by the wives of unfaithful husband as punishment, and rarely in some cases of masturbatory catastrophe or neglected priapism.

The first documented report of macroscopic penile replantation was reported in 1929 by Ehrich, and since then there were reports of at least 30 cases of penile autoamputation with successful reimplantation. There are several factors that determine the outcomes which include the time of presentation from the injury vis‐a‐vis, the warm ischemic time as it happened in the case of our patient who presented about 30 h after injury with the penis mummified precluding reimplantation of the amputated penis. Other factors include are the degree of injury, type of injury, the availability of the necessary instruments, and the expertise of the surgeon.

Our patient presented 30 h after injury which is quite late for the penile warm ischemic time as a result of that he he could not benefit from reimplantation. If the patient was presented early, the amputated part should be washed in saline, wrapped in clean gauze piece, and placed in saline ice slush within a sterile plastic bag, as reported by Adigun et al. Omar Riyach et al. reported a successful replantation of an incomplete amputated penis of a 35‐yearold Berber using a macrosurgical technique, whereas Mohammed Reza et al. reported a successful macrosurgical reimplantation of a complete penile amputation in a 30‐year‐old man who presented 8 h after injury.

Our patient was presented with an associated scrotal laceration exposing the testicles, as opposed to the patient reported by Obi et al. were the scrotum and the testicles were not involved. Adigun et al. also reported an intact scrotum and testicles in the patient as they reported that the patient was presented with traumatic penile amputation from grinding machine.

In the patient reported by Adigun et al., there were no other associated injuries noted, but in our patient, he had associated frictional burns in addition to the traumatic penile amputation.

## Conclusion

Traumatic penile amputation is a urological emergency which cause a lot of concern and anxiety. The etiology varies but ranges from self‐infliction in psychiatric patients, to industrial accidents or assaults, and rarely from circumcision mishaps, especially guillotine type.

Whenever it happened, it can be devastating to the patient, his spouse, his relatives, and very tasking to the urologist managing the case, especially when the stump was brought late after 24 h, and unpreserved, as is the case of our patient. If it happens, there is necessity for early urological referral and the amputated part should be wrapped in saline ice slush and placed in normal saline to preserve viability, so that it can be replanted successfully.

## Conflict of Interest

None declared.
